# Effects of *Conocarpus lancifolius* and crocin on superoxide dismutase and protein kinase B genes and protein expressions in diabetic rats

**DOI:** 10.1371/journal.pone.0326676

**Published:** 2025-06-26

**Authors:** Mohamed Lotfy, Abdallah Khalaf, Faisal AlBarghouthy, Abdulla Alhashmi, Hesham Adam, Abdulla Almesmari, Biduth Kundu, Taoufik Ksiksi

**Affiliations:** Biology Department, College of Science, United Arab Emirates University, Al Ain, United Arab Emirates; American University of Madaba, JORDAN

## Abstract

**Background:**

Diabetes mellitus, characterized by chronic hyperglycemia, is a global health challenge. Effective management strategies often focus on improving insulin sensitivity and enhancing antioxidant defenses to mitigate diabetes-related complications. This study explored the therapeutic potential of *Conocarpus lancifolius* (Co) and crocin in modulating insulin signaling and antioxidant gene expression in diabetic rats.

**Methods:**

Male Wistar rats were rendered diabetic via alloxan injection and treated orally with *C. lancifolius* and crocin. Control groups included normal healthy control and untreated diabetic rats. Insulin signaling pathways were assessed by measuring key markers such as protein kinase B (AKT) through western blot and RT-PCR analysis. Antioxidant gene expressions, including superoxide dismutase (SOD), were quantified to evaluate oxidative stress response.

**Results:**

*C. lancifolius* and crocin treatment significantly improved fasting blood-glucose levels and insulin sensitivity in diabetic rats. Notably, *C. lancifolius* and crocin administration resulted in the upregulation of AKT, promoting glucose uptake in peripheral tissues. Additionally, *C. lancifolius* and crocin increased the expression of the SOD gene, improving antioxidant defense in diabetic rats.

**Discussion:**

*C. lancifolius* and crocin had a dual beneficial effect on diabetic rats by modulating key components of the insulin signaling pathway and bolstering antioxidant defenses. The upregulation of AKT as a key gene in insulin signaling pathways suggests an improvement in insulin sensitivity, which is crucial for glycemic control. Concurrently, the elevated antioxidant SOD gene expression indicates reduced oxidative stress, which is vital for preventing diabetic complications.

**Conclusion:**

Our findings demonstrate that *C. lancifolius* and crocin significantly improved glucose tolerance, reduced fasting blood glucose levels, and enhanced antioxidant defense in diabetic rats. Histopathological improvements in the pancreas, liver, kidney, and heart further highlight their protective effects against diabetes-induced tissue damage. Additionally, the upregulation of SOD and AKT at both gene and protein levels suggests a role in modulating oxidative stress and insulin signaling pathways. While promising, further studies are needed to clarify their molecular mechanisms, particularly regarding insulin receptor interaction. These findings support the use of *C. lancifolius* and crocin as natural adjunct therapies to enhance diabetes treatment outcomes.

## 1 Introduction

Diabetes mellitus (DM) is a chronic metabolic disorder characterized by persistent hyperglycaemia resulting from impaired insulin secretion, insulin resistance, or both, leading to disturbances in carbohydrate, lipid, and protein metabolism [1,2]. It is primarily classified into type 1, type 2, and gestational diabetes, with type 2 accounting for approximately 90% of all cases [3–5]. If left untreated, DM can result in severe complications such as cardiovascular disease, nephropathy, neuropathy, and retinopathy [6]. Although several pharmacological options exist, including insulin secretagogues, insulin sensitizers, and glucose absorption inhibitors [7,8], current therapies often fail to achieve long-term glycaemic control and are associated with adverse effects such as hypoglycaemia, weight gain, gastrointestinal discomfort, and increased cardiovascular risk [9–14]. Consequently, there is a growing shift toward natural therapies due to their perceived safety and better patient tolerance [15,16].

Oxidative stress plays a central role in the pathogenesis of diabetes and its complications by damaging pancreatic β-cells, impairing insulin signalling, and promoting systemic inflammation. Chronic hyperglycaemia increases reactive oxygen species (ROS) production and overwhelms the body’s antioxidant defence mechanisms, further exacerbating insulin resistance and cellular damage [17]. Therefore, targeting oxidative stress pathways has emerged as a promising strategy in diabetes management. In this context, medicinal plants rich in antioxidants have garnered considerable attention for their potential to modulate oxidative stress, improve glucose homeostasis, and alleviate diabetic complications.

Historically, a wide variety of plants have been used for medicinal purposes. Plant extracts often exhibit anti-inflammatory and antioxidant properties, which are beneficial for managing chronic conditions such as diabetes, atherosclerosis, neurodegenerative diseases, and cancer [18]. These natural therapies can reduce oxidative stress and inflammation, improve insulin sensitivity, and support overall metabolic health [19–21].

*Conocarpus lancifolius* (Damas), an evergreen tree native to Somalia and Yemen and widely cultivated in the Gulf region, has been traditionally used to treat gastrointestinal and respiratory disorders [22]. Phytochemical analyses have identified various bioactive compounds in its extracts, including flavonoids, phenolic acids, tannins, and ellagic acid derivatives, known for their antioxidant and anti-inflammatory effects [23–26]. A few studies have reported the plant’s ability to inhibit α-glucosidase and α-amylase enzymes, supporting its potential antidiabetic effects [22,24,27]. Despite these findings, prior research has largely focused on the plant’s general pharmacological profile or in *vitro* enzyme inhibition, with limited in vivo and molecular data. While some reports suggest, antioxidant, anticancer, antiinflamatory and antidiabetic potential [28–31], but the direct molecular effects of *C. lancifolius* on insulin signaling or oxidative stress pathways and the modulation of genes such as AKT and SOD, which play vital roles in glucose homeostasis and oxidative stress regulation, remains underexplored.

In parallel, crocin, a major carotenoid of *Crocus sativus* (saffron), is well documented for its therapeutic potential in diabetes. It protects pancreatic β-cells, improves insulin secretion, reduces oxidative stress, and enhances glucose and lipid metabolism [19,20,32–35]. Crocin also modulates insulin signaling pathways and has shown promise in reducing diabetic complications [36–39]. However, most studies use crocin in limited molecular evaluations.

Protein kinase B (AKT) is a key molecule in the insulin signaling pathway, promoting glucose uptake, insulin sensitivity and glycogen synthesis. Impairments in AKT signaling contribute to insulin resistance and hyperglycemia [40–42]. On the other hand, superoxide dismutase (SOD) is a central antioxidant enzyme that protects cells from reactive oxygen species (ROS), which are elevated in diabetes and contribute to β-cell dysfunction and complications [43–45].

The current study addresses these gaps using comprehensive in *vivo* approaches, including histopathological examination, immunofluorescence imaging, PCR, and western blotting to assess the effects of *C. lancifolius* extract and crocin on diabetic conditions. Predominantly, this study aims to evaluate the molecular effects of *C. lancifolius* and crocin in a diabetic rat model, with a focus on gene expression of AKT and SOD, alongside tissue-level changes. By employing a multifaceted experimental design, we seek to provide new insights into the mechanisms through which these natural agents may alleviate diabetic pathogenesis.

## 2 Materials and methods

### 2.1 Plant material and extract preparation

Fresh young leaves of *C. lancifolius* were gathered from Al-Ain, United Arab Emirates (N24.2 E55.6) around October 2023. The aerial samples of *C. lancifolius* leaves were verified by a plant taxonomist and deposited at the Herbarium of the Department of Biology, College of Science, United Arab Emirates University, Al-Ain. A voucher specimen (Herbarium voucher number UAEU-NH014704) was submitted to the botanical herbarium of the UAE University. Preparation of *C. lancifolius* crude extract was using fresh leaves of *C. lancifolius* were collected, washed thoroughly, and cut into small segments. The leaves were then dried in an oven at 60°C for 7 days until completely dehydrated. The dried material was ground into a fine powder using a high-speed Mill herb grinder (Great Wall, China). A total of 1 kg of the powdered leaves was extracted through the use of a using the simple maceration method. Specifically, the powder was soaked in 1200 mL of methanol in tightly sealed containers and agitated on an orbital shaker at 100 rpm for 72 hours at room temperature. The mixture was then filtered using Whatman No. 1 filter paper, and the resulting filtrate was concentrated under reduced pressure using a rotary evaporator at approximately 40°C (Stuart, UK). This process yielded 18.3 g of crude *C. lancifolius* extract, which was stored in amber-colored bottles at −20°C until use. Phytochemical screening of the extract confirmed the presence of free and bound anthraquinones, saponins, flavonoids, and tannins, while alkaloids were absent [46].

### 2.2 Experimental animals

This study used male Wistar rats weighing about 200 g, sourced from the Animal House Facility of College of Medicine and Health Sciences, United Arab Emirates University. The rats were kept in metal cages under standardized environments, including room temperature (25°C), humidity, and a 12-h light-dark round, with free access to food and ad libitum water consumption [47]. Ethical approval for the experiment was granted by the Animal Ethics Committee of the United Arab Emirates University (Approval number ERA_2023_3067).

### 2.3 Induction of diabetes and experimental grouping

Experimental diabetes was induced in male rats by a single intraperitoneal (IP) injection of alloxan monohydrate (Sigma, USA) at a dose of 120 mg/kg body weight, dissolved in sterile 0.9% saline. Five days post-injection, and following an 18-hour overnight fast, blood glucose levels were measured via the tail vein. Rats with fasting blood glucose levels ≥200 mg/dL were considered diabetic, consistent with previously established criteria [48].

Upon confirmation of diabetes, animals were randomly assigned to four groups (n = 6 per group):

(1) Normal untreated control (C),
(2) Diabetic untreated (D),
(3) Diabetic treated with *C. lancifolius* extract (DCoT),
(4) Diabetic treated with crocin (DCrT).

All animals received daily oral treatments via gavage for four weeks. Rats in the control and diabetic untreated groups received 1 mL of sterile 0.9% saline, which also served as the vehicle for the active treatment groups. Diabetic rats in the DCoT group received *C. lancifolius* extract at 500 mg/kg body weight, while those in the DCrT group received crocin at 100 mg/kg body weight. Both treatment compounds were freshly prepared each day by dissolving the required dose in sterile 0.9% saline and administered in a total volume of 1 mL.

To enhance consistency in drug absorption and reduce variability in pharmacokinetic response, a standardized 12-hour fasting period was implemented prior to each gavage administration. This protocol aligns with established practices in preclinical studies to control food-related absorption variability while minimizing fasting-induced physiological stress [49].

Dosage selection for both *C. lancifolius* and crocin was informed by prior safety and efficacy studies. Acute toxicity assessments of *C. lancifolius* indicated no signs of toxicity or mortality in mice receiving oral doses up to 2000 mg/kg, suggesting a favorable safety profile [24,50]. Similarly, crocin has demonstrated low toxicity in rodents, with no mortality observed at oral or intraperitoneal doses up to 3 g/kg in acute and sub-acute toxicity studies [51,52]. Nevertheless, further studies are warranted to evaluate the long-term safety of these compounds, particularly regarding their potential effects on hepatic and renal function during chronic use.

### 2.4 Intraperitoneal glucose tolerance test

Intraperitoneal glucose tolerance test (IPGTT) was performed on the rats after the four-week treatment. Following an 18-h overnight fast, each rat received an intraperitoneal (i.p.) injection of glucose at 2 g/kg body weight, as per a previously documented protocol [53]. Blood-glucose levels were measured from the tail vein using a glucometer (OneTouch Ultra, LifeScan, PA, USA) at 0 (pre-glucose load), 30, 60, 120, and 180 min after glucose administration.

### 2.5 Blood collection and tissue processing

At the end of the experiments, all rats were anesthetized with intraperitoneal injections of ketamine hydrochloride (90 mg/kg) and xylazine hydrochloride (10 mg/kg) [54]. Following decapitation, blood samples were collected for biochemical analysis. The rats were dissected, and pancreas, liver, kidney, and heart tissues were rapidly removed for histological analysis. Liver tissues were also collected for molecular biological investigation.

### 2.6 Serum superoxide dismutase estimation

Upon completion of the study, the activity of serum SOD was measured using a kit from Sigma-Aldrich (MI, USA) according to the manufacturer’s protocol. SOD activity, expressed as a percentage of inhibition rate, was evaluated using an indirect assay method involving xanthine oxidase and detected changes in a color reagent. Total protein concentration was determined using the quick start™ Bradford protein assay kit from Bio Rad (CA, USA), which relies on changes in reaction color to quantify the total protein concentration within the serum samples. SOD and total protein levels were then calculated for each group as SOD-specific activity units per milligram of protein [55].

### 2.7 Tissue histological analysis

The pancreas, liver, kidney, and heart tissues were dissected into 3–4 mm^3^ fragments, rinsed in phosphate-buffered saline, and then fixed in a 10% formalin solution. The tissues were then dehydrated through a series of increasing ethanol concentrations (50%–100%), cleared with xylene, and embedded in paraffin wax. Tissue blocks were sectioned into 5 µm thick slices using a microtome (Leica 2135, Germany) and stained with hematoxylin and eosin (H&E) [56]. Images for histological examination were captured using a light microscope (Zeiss Oberkochen, Germany), and tissue morphology was analyzed with Image J^®^ software (NIH, Bethesda, Maryland, USA). Histological evaluations were performed blindly, without prior knowledge of the respective groups.

### 2.8 Immunofluorescence analysis

The pancreatic tissue fragments were initially fixed in 10% formalin fixative, embedded in paraffin wax, and then prepared for immunofluorescence following a formerly documented protocol [57]. Insulin and glucagon were detected immunologically using specific antibodies. For insulin, a primary rabbit monoclonal antibody (ab181547, Abcam, MA, USA, dilution 1:1000) was linked to a secondary FITC-labeled goat anti-rabbit IgG antibody (ab6717, Abcam, MA, USA, dilution 1:1000). For glucagon detection, a primary mouse monoclonal IgG antibody (sc-514592, Santa Cruz Biotechnology, TX, USA, dilution 1:1000) was paired with a secondary TRITC-labeled donkey anti-mouse IgG antibody (ab6817, Abcam, MA, USA, dilution 1:1000). Immunoreactive insulin beta cells and glucagon alpha cells were visualized using an Olympus fluorescence microscope (Hamburg, Germany). For each group, three immunofluorescence images were captured per rat for subsequent analysis.

### 2.9 Quantitative real-time PCR

Liver tissues from rats were employed to assess mRNA transcript levels of AKT and SOD genes using quantitative real-time PCR (qRT-PCR). Each experimental group of rats had their samples preserved in RNAlater and stored at −80°C. Total RNA was then isolated by homogenizing the liver tissues from each sample using the RNeasy Protect kit (Qiagen, TX, USA). Following this, cDNA synthesis was performed using a high-capacity cDNA reverse transcription kit from Thermo Fisher Scientific (MA, USA). Quantitative PCR analysis was conducted on an Applied Biosystems QuantStudio-5 Real-Time PCR System. The PowerUp SYBR Green detection method (Thermo Fisher Scientific) was employed for qPCR, utilizing specific primers for the target genes. Average cycle threshold (Ct) values were used to assess the relative expression differences between the four groups of rats. The relative expression of the target genes was normalized to the internal control, glyceraldehyde 3-phosphate dehydrogenase (GAPDH), for each gene. Relative changes in gene expression were calculated using the ΔΔCt (threshold cycle) method, with fold-change values derived from the equation 2^−ΔΔCt^ as previously described [58]. Primer sequences as previously described [48] were used for the qRT-PCR experiment are detailed in Table 1.

**Table 1:** Primer sequences used for qRT-PCR.

Gene	Forward Primer (5’ → 3’)	Reverse Primer (5’ → 3’)
SOD	CCGGTGCAGGGCGTC	TCCTGTAATCTGTCCTGACACCA
AKT	TGAGACCGACACCAGGTATTTTG	GCTGAGTAGGAGAACTGGGGAAA
GAPDH	GGCACAGTCAAGGCTGAGAATG	ATGGTGGTGAAGACGCCAGTA

### 2.10 Western blot analysis

Liver tissues from rats were homogenized in Radio-immunoprecipitation assay lysis buffer (Thermo Fisher Scientific, Illinois, USA) on ice using a tissue homogenizer (Omni International, GA, USA). The homogenates were centrifuged at 13,000 × g for 20 min at 4°C, and the resulting supernatants were collected to determine protein concentrations using a protein assay kit based on the Bradford method, following the manufacturer’s instructions (Bio Rad, California, USA). Tissue lysates were mixed with an equal volume of 2X loading buffer and then subjected to electrical separation on an SDS-polyacrylamide gel. The resulting protein bands were transferred onto a polyvinylidene difluoride membrane using a transfer buffer saline (TBS) at 4°C for 1 h [59]. The membrane was blocked for 1 h at room temperature with a blocking solution containing 5% non-fat milk in TBS and then incubated overnight at 4°C with one of the following primary antibodies: AKT Rabbit monoclonal antibody (ab179463, Abcam, MA, USA, dilution 1:10,000), SOD rabbit recombinant antibody (ab51254, Abcam, MA, USA, dilution 1:50,000), or anti-GAPDH Rabbit Recombinant antibody for housekeeping control (ab181602, Abcam, MA, USA, dilution 1:10,000). After primary antibody incubation, the membrane was incubated for 1 h with horseradish peroxidase-conjugated goat anti-rabbit IgG secondary antibody (65–6120, Invitrogen, MA, USA, dilution 1:10,000). All antibodies were diluted in blocking buffer [48]. Target protein band densities were visualized using the enhanced chemiluminescence system (32106, Thermo Scientific, MA, USA), and the blot images were captured using a CCD camera. The relative expression levels of the proteins were normalized to the housekeeping protein GAPDH and quantified using Image Lab 4.1 software (Figs S1–S4).

### 2.11 Statistical analysis

Data were expressed as mean plus/minus standard error of the means (SEM). Statistical analyses were conducted using SPSS version 15.0 (IBM Corporation, Armonk, NY, USA). One-way analysis of variance (ANOVA) was performed to evaluate differences among multiple groups, followed by Tukey’s post hoc test. A significance threshold level of P ≤ 0.05 was used for all analyses. Furthermore, corrections for multiple comparisons were applied through the post-hoc test to ensure result reliability. In the figures, hash symbols (#) indicate significant differences between the diabetic untreated group and the normal untreated control group, while asterisks symbols (*) indicate significant differences between diabetic treated groups and the diabetic untreated control group.

## 3 Results

### 3.1 Effect of *C. lancifolius* and crocin treatment on intraperitoneal glucose tolerance test and fasting blood-glucose levels

**Fig 1:**
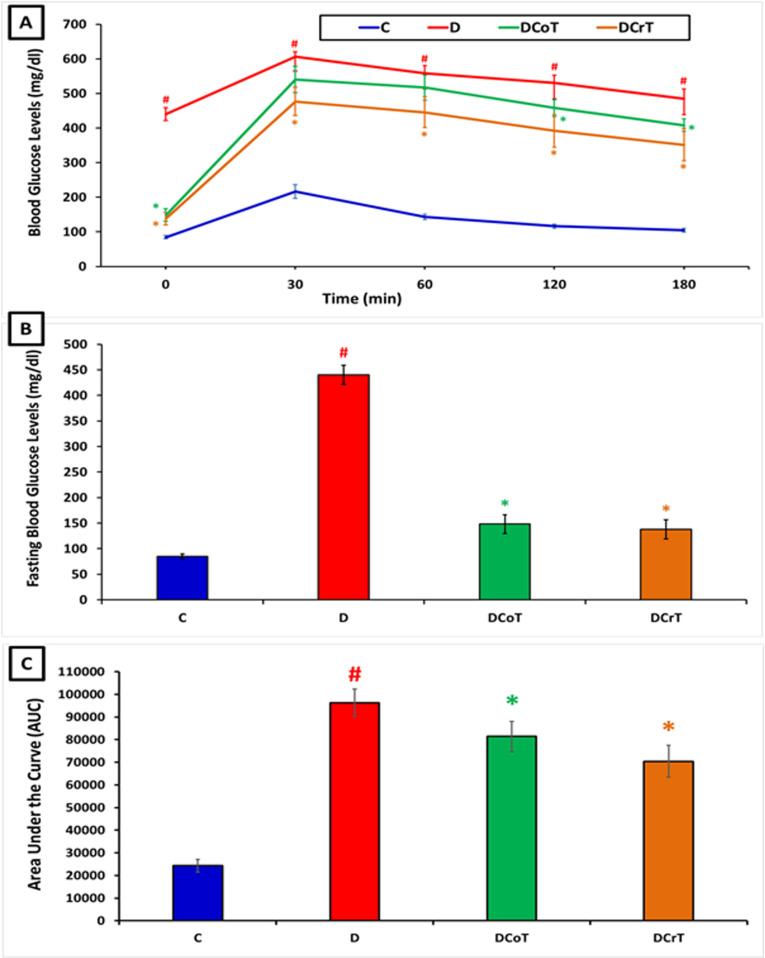
[A] Illustrates blood-glucose levels measured during the intraperitoneal glucose tolerance test (IPGTT) conducted at the conclusion of the experiment. [B] Displays the fasting blood-glucose levels recorded at the end of the study. [C] Shows the areas under the curve (AUC) for all the rat groups. The values are presented as mean ± standard error of the mean (SEM). ^#^ Indicates significant differences (P ≤ 0.05) between the untreated diabetic group (D) and the control group (C). *Indicates significant differences (P ≤ 0.05) between the C. lancifolius-treated group (DCoT) or crocin-treated diabetic group (DCrT) and untreated diabetic group (D). Each group consisted of six rats (n = 6).

The IPGTT results showed significant reductions (P ≤ 0.05) in blood-glucose levels for the normal control (C), *C. lancifolius*-treated diabetic group (DCoT), and crocin-treated diabetic group (DCrT), compared to that for the untreated normal control (C) and untreated diabetic groups (D) at 0, 60, 120, and 180 min post glucose administration (Fig 1A). Additionally, fasting blood-glucose levels were significantly reduced in the diabetic group compared to that in the untreated control group (P ≤ 0.05). Both *C. lancifolius* and crocin treatments in diabetic groups resulted in a significant decrease in fasting blood-glucose levels compared to the untreated diabetic group (P ≤ 0.05) (Fig 1B). Moreover, diabetic rats exhibited significantly impaired glucose tolerance (P ≤ 0.05), characterized by higher blood-glucose levels and a greater area under the glucose curve (AUC) compared to normal control rats. Treatment with *C. lancifolius* and crocin in diabetic rats significantly reduced blood-glucose levels (P ≤ 0.05) and the AUC, indicating that *C. lancifolius* and crocin improve glucose tolerance in diabetic rats (Fig 1C).

### 3.2 Effect of *C. lancifolius* and crocin treatment on serum SOD levels

**Fig 2:**
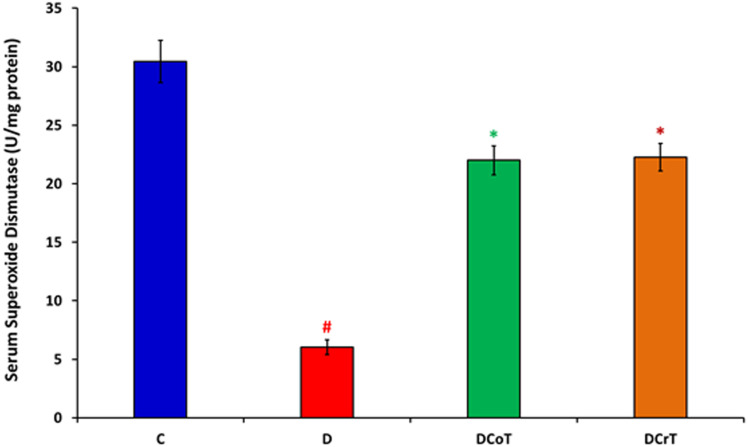
Serum superoxide dismutase levels at the end of the study. The values are presented as mean ± SEM. ^#^ Indicates significant differences (P ≤ 0.05) between the untreated diabetic group (D) compared to the control group (C). *Indicates significant differences (P ≤ 0.05) between the C. lancifolius-treated group (DCoT) or crocin-treated diabetic group (DCrT) and the untreated diabetic group (D). Each group consisted of six rats (n = 6).

The impact of crocin treatment on serum SOD levels revealed that diabetic untreated rats displayed significantly lower levels of the endogenous antioxidant enzyme SOD compared to normal control rats (P ≤ 0.05). Crocin intervention effectively restored serum SOD enzyme levels, indicating its antioxidant efficacy. Moreover, diabetic rats treated with *C.lancifolius-*treated, and crocin showed increased serum SOD levels compared to the untreated diabetic group. This increase was significantly pronounced (p ≤ 0.05) in the diabetic rats treated with *C. lancifolius* and crocin compared to the untreated diabetic group (Fig 2).

### 3.3 Effect of *C. lancifolius* and crocin treatment on pancreas, liver, kidney, and heart histopathology

**Fig 3:**
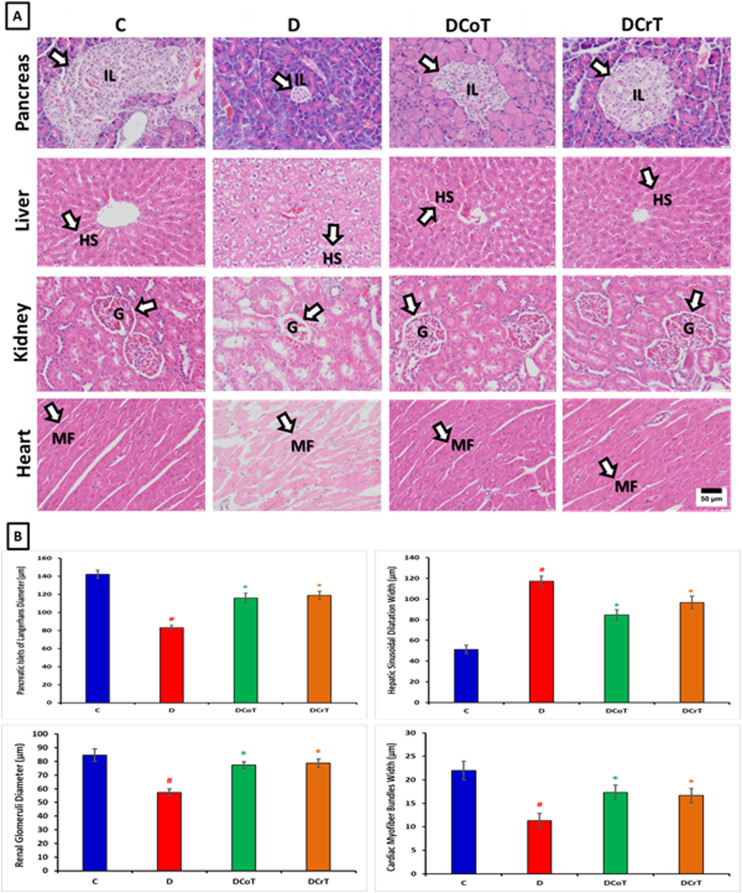
[A] Histopathological images of pancreas, liver, kidney, and heart tissues from the studied rat groups, stained with H&E. The groups include control (C), untreated diabetic (D), C. lancifolius-treated (DCoT), and diabetic crocin-treated (DCrT) rats. Measurements were conducted using ImageJ software. Parameters included pancreatic Islets of Langerhans maximum diameter (µm), hepatic sinusoidal width (µm), renal glomerular maximum diameter (µm), and cardiac myofiber bundles width (µm), measured from randomly selected, clearly defined structures per tissue section. Pancreas: Untreated diabetic rats show a reduction in the size of pancreatic islets of Langerhans (IL), correlated with a decrease in insulin-secreting beta cells. In diabetic-treated rats, the IL size is nearly restored to normal. Liver: Untreated diabetic rats exhibit significant dilation and congestion of the hepatic central vein, along with hepatic sinusoidal dilatation (HS), indicating vascular stasis and congestion in the hepatic parenchyma. In treated groups, the congestion is reduced, and the structure is more normalized. Kidney: Untreated diabetic rats show severe distortion of glomerular architecture (G) and necrotic changes, with many convoluted tubules displaying hypertrophic cells and vacuolization. Diabetic-treated groups, however, exhibit nearly normal renal morphology, with clearly visible glomeruli, intact basement membranes, and mesangial tissue. Heart: Untreated diabetic rats show scattered cardiac muscle fibers, with disorganized cardiac myofibers (MF). In diabetic-treated rats, there is significant improvement in the orientation and size of myofibers, with normal acidophilic sarcoplasm and centrally located nuclei. [B] display histograms depicting the different parameters counts, of pancreatic, hepatic, renal, and cardiac tissues respectively, following C. lancifolius and crocin treatment. The values are presented as mean ± SEM. ^#^ Indicates significant difference (P ≤ 0.05) between the untreated diabetic group and the control group. * Indicates a significant difference (P ≤ 0.05) between the C. lancifolius-treated or crocin-treated diabetic group and the untreated diabetic group. Each group consisted of six rats (n = 6). 400x magnification, scale bar 50 μm.

The pancreatic, liver, kidney, and heart tissue samples from rats were subjected to H&E staining and examined under a light microscope to assess any morphological alterations. In pancreatic tissue samples from normal untreated control rats, the pancreas exhibits a typical histological structure characterized by well-defined lobules composed of acini and pancreatic islets (islets of Langerhans). The acini, arranged in clusters, are responsible for producing digestive enzymes. Meanwhile, the pancreatic islets are distributed throughout the parenchyma and contain beta and alpha cells, which produce insulin and glucagon, respectively. In diabetic untreated rats, histological changes included reduced size and number of islets, as well as vacuolation and degeneration of acinar cells due to chronic hyperglycemia. Treatment with *C. lancifolius* and crocin in diabetic rats resulted in the restoration of normal islet morphology and reduced acinar degeneration, attributed to the antioxidant and anti-inflammatory properties of crocin (Fig 3).

Liver tissue samples in normal untreated control rats showed a characteristic histological organization, with well-defined lobules composed of hepatocytes arranged radially around a central vein. Hepatocytes are cuboidal cells with prominent nuclei and eosinophilic cytoplasm, responsible for numerous metabolic functions, including glycogen storage, bile production, and detoxification. In diabetic untreated rats, histological changes included hepatocyte swelling, lipid accumulation, and fibrosis due to insulin resistance and dysregulated glucose metabolism. In diabetic *C. lancifolius* and crocin-treated rats, there was a reduction in hepatocyte swelling and lipid accumulation, along with improved liver architecture because of the antioxidant and anti-inflammatory properties of *C. lancifolius* and crocin (Fig 3).

In the kidney tissue samples from normal untreated control rats, the histological structure appeared typical, with the outer renal cortex containing glomeruli and proximal and distal convoluted tubules, while the medulla contained loops of Henle and collecting ducts. Glomeruli are composed of capillary loops surrounded by Bowman’s capsule, where filtration of blood occurs. In diabetic untreated rats, histological changes included glomerular hypertrophy, mesangial expansion, and tubular atrophy due to diabetic nephropathy. In diabetic *C. lancifolius-*treated rats and crocin-treated rats showed attenuation of glomerular hypertrophy and mesangial expansion and preservation of tubular integrity due to the antioxidant and anti-inflammatory effects of crocin (Fig 3).

The heart tissue samples from untreated control rats exhibited a characteristic histological structure, with cardiac muscle fibers organized into myocardial bundles. Adjacent cardiac muscle cells were interconnected by intercalated discs, which facilitated synchronized contraction. In diabetic untreated rats, histological changes included cardiomyocyte hypertrophy and interstitial fibrosis due to diabetic cardiomyopathy. In diabetic *C. lancifolius-*treated rats and crocin-treated rats there was mitigation of cardiomyocyte hypertrophy and fibrosis due to the antioxidant and anti-inflammatory effects of crocin (Fig 3).

### 3.4 Effect of *C. lancifolius* and crocin treatment on the number of insulin- and glucagon-immuno-positive cells in the pancreas

**Fig 4:**
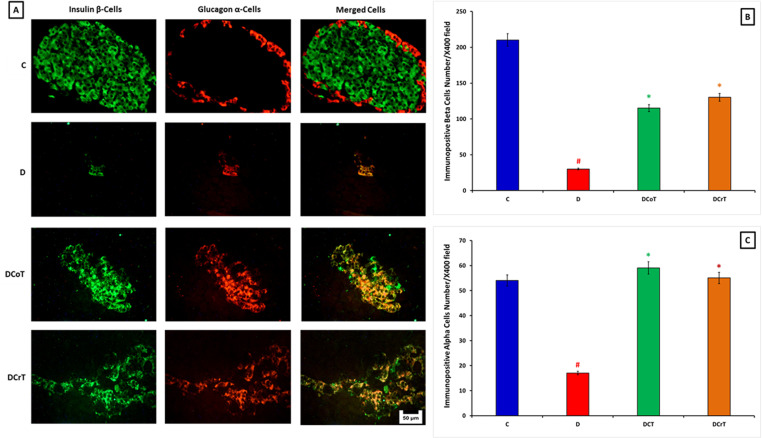
[A] Immunofluorescence double-labeling images illustrate insulin-immunoreactive cells (green) and glucagon-positive cells (red) in the islets of the control (C), untreated diabetic (D), C. lancifolius-treated (DCoT), and diabetic crocin-treated (DCrT) groups. The figure shows a more pronounced presence of insulin-positive cells in the islets of crocin-treated diabetic rats compared to untreated diabetic rats. [B] and [C] display histograms depicting the counts of insulin and glucagon immunoreactive cells, respectively, following C. lancifolius and crocin treatment. Notably, insulin- and glucagon-positive cell counts significantly increased after C. lancifolius and crocin treatment. The values are presented as mean ± SEM. ^#^ Indicates significant difference (P ≤ 0.05) between the untreated diabetic group and the control group. * Indicates a significant difference (P ≤ 0.05) between the C. lancifolius-treated or crocin-treated diabetic group and the untreated diabetic group. Each group consisted of six rats (n = 6). 400x magnification, scale bar 50 μm.

The immunofluorescence analysis demonstrated notable alterations in the quantity of insulin- and glucagon-positive cells in the pancreas of rats following crocin treatment. Specifically, crocin administration increased insulin-positive beta cells in the pancreatic islets of normal control, diabetic *C. lancifolius*-treated, and DCrT rats compared to untreated diabetic rats. This suggests that crocin treatment supports the proliferation or preservation of insulin-secreting beta cells. In contrast, there was a slight decrease in the number of glucagon-positive alpha cells within the pancreatic islets of normal control, diabetic *C. lancifolius*-treated, and DCrT rats compared to untreated diabetic rats. This implies that crocin treatment may exert an inhibitory effect on glucagon-secreting alpha cells (Fig 4A). These trends are shown in Fig 4B and 4C, highlighting significant (P ≤ 0.05) changes in the quantities of insulin- and glucagon-positive cells, respectively, in untreated diabetic, diabetic *C. lancifolius*-treated, and DCrT rats compared to normal untreated controls. These findings further underscore the beneficial effects of crocin treatment on pancreatic endocrine cells in diabetic rats.

### 3.5 Effect of *C. lancifolius* and crocin treatment on the gene expression of SOD and AKT

**Fig 5:**
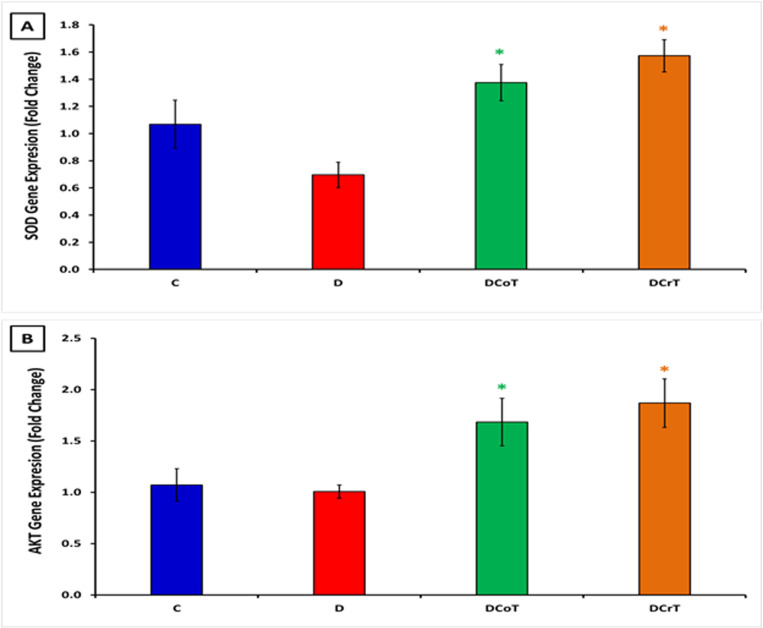
Gene expression fold-change values obtained from qPCR analysis. [A] Alterations in superoxide dismutase (SOD) gene expression. [B] shows changes in protein kinase B (AKT) gene expression in the liver tissue of rats. The data were normalized to the housekeeping gene glyceraldehyde-3-phosphate dehydrogenase (GAPDH) and plotted relative to equivalent tissues from normal control and untreated diabetic rats. The values are presented as mean ± SEM. ^#^ Indicates a significant difference (P ≤ 0.05) between the untreated diabetic group and the control group. * Indicates a significant difference (P ≤ 0.05) between the C. lancifolius-treated or crocin-treated diabetic group compared to untreated diabetic group. Each group consisted of six rats (n = 6).

Treatment with *C. lancifolius* and crocin had significant effects on the gene expression of SOD and AKT. There was a significant increase in SOD gene expression in the *C. lancifolius*-treated and crocin-treated diabetic rats compared to that in untreated diabetic rats (P ≤ 0.05) (Fig 5A). This finding suggests that *C. lancifolius* and crocin effectively enhance the expression of SOD. Additionally, AKT gene expression significantly increased in *C. lancifolius*-treated and crocin-treated diabetic rats compared to that in the untreated diabetic group (P ≤ 0.05) (Fig 5B). This finding indicates that these treatments may positively influence the AKT signaling pathway.

### 3.6 Effect of *C. lancifolius* and crocin treatment on the protein expression of SOD and AKT

**Fig 6:**
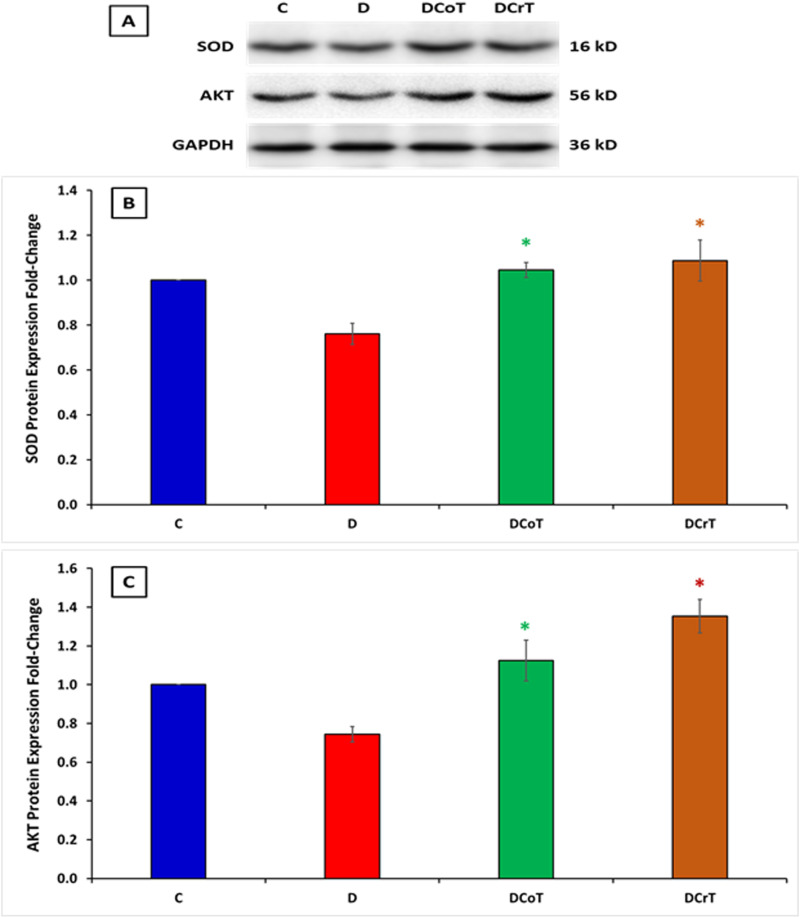
[A] Western blot analysis of superoxide dismutase (SOD) and protein kinase B (AKT) in the liver tissues of normal, non-diabetic control rats, and diabetic rats treated with crocin. Glyceraldehyde-3-phosphate dehydrogenase (GAPDH) was used as the housekeeping protein to normalize protein levels. Quantified protein expressions of SOD [B] and AKT [C]. The values are presented as mean ± SEM. ^#^ Indicates a significant difference (P ≤ 0.05) between the untreated diabetic group and the control group. * Indicates a significant difference (P ≤ 0.05) between the C. lancifolius-treated or crocin-treated diabetic group and the untreated diabetic group. Each group consisted of six rats (n = 6).

In addition to evaluating the gene expression levels of SOD and AKT in the liver in all rats, we conducted further analysis to assess their protein expressions. Densitometric analysis of the western blot data demonstrated a significant increase in SOD protein expression in the *C. lancifolius*-treated and crocin-treated diabetic rats compared to the untreated diabetic rats (P ≤ 0.05). This suggests that crocin treatment leads to a notable augmentation in SOD protein expression, indicating enhanced antioxidant activity in the liver. Similarly, crocin treatment significantly increased AKT protein expression in the *C. lancifolius-*treated and crocin-treated diabetic rats compared to the untreated diabetic rats (P ≤ 0.05). This finding implies that crocin treatment may promote the upregulation of AKT protein expression, potentially influencing various cellular processes, including glucose metabolism and cell survival. Fig 6A–C depict the densitometric analysis outcomes of the western blot.

## 4 Discussion

The primary objective of this study was to evaluate the therapeutic potential of *C. lancifolius* extract and crocin in improving glucose metabolism, enhancing insulin signaling, alleviating oxidative stress, and preserving organ integrity in alloxan-induced diabetic rats. Specifically, we aimed to investigate the modulatory effects of these compounds on the PI3K/AKT insulin signaling axis, antioxidant defense via superoxide dismutase (SOD) activity, and their protective roles in preserving the structure of vital organs such as the liver, pancreas, kidneys, and heart.

Our findings indicate that *C. lancifolius* extract exerts significant antidiabetic effects. Diabetic rats treated with the extract showed improved glucose regulation, increased hepatic AKT gene expression, and enhanced SOD activity, suggesting a dual role in modulating insulin signaling and combating oxidative stress. These outcomes are supported by histopathological evidence showing preservation of pancreatic islets, reduced hepatic vacuolization, normalized glomerular structures in kidneys, and improved cardiac myofiber organization. These structural improvements align with molecular findings and emphasize the extract’s therapeutic potential.

The novel findings of the study include a significant increase in AKT activation and superoxide dismutase (SOD) activity in the liver, kidney, and pancreas of treated rats, accompanied by improved tissue morphology and biochemical profiles. These results support the therapeutic potential of *C. lancifolius* extract and crocin in ameliorating diabetic complications via modulation of insulin signaling and oxidative stress pathways.

The observed effects of *C. lancifolius* can be attributed to its rich phytochemical content, particularly flavonoids, tannins, and phenolic acids, which are known for their antioxidants and insulin-sensitizing properties [60,61]. Previous studies have shown that *C. lancifolius* enhances antioxidant defenses under environmental stress conditions, such as salinity, by upregulating SOD expression [62]. In our study, we report for the first time that *C. lancifolius* significantly upregulates hepatic AKT gene expression in diabetic rats, indicating activation of the PI3K/AKT pathway—an essential mediator of insulin-stimulated glucose uptake [63]. This pathway enhances translocation of glucose transporter 4 (GLUT4) to the cell membrane, thereby improving glucose clearance.

Moreover, the upregulation of SOD gene expression by *C. lancifolius* may involve modulation of the nuclear factor erythroid 2–related factor 2 (Nrf2) signaling pathway. Nrf2 is a master transcriptional regulator of antioxidant responses, controlling genes that encode enzymes like SOD and catalase. Activation of the Nrf2 pathway protects tissues from reactive oxygen species (ROS)-induced injury, which is especially important in hyperglycemic conditions [64]. Also, the ability of *C. lancifolius* to prolong lifespan in *Caenorhabditis elegans* through antioxidant mechanisms and purine metabolism regulation, further corroborating its bioactivity [65].

In parallel, crocin treatment produced comparable beneficial effects in diabetic rats. Crocin significantly improved glucose tolerance, reduced blood glucose levels, and upregulated hepatic AKT gene expression, suggesting enhanced insulin sensitivity through activation of the PI3K/AKT cascade. Histological analysis revealed preservation of pancreatic islets, hepatocellular architecture, renal glomeruli, and cardiac tissue integrity in crocin-treated animals.

Crocin’s antidiabetic effects are well-documented and involve multiple complementary mechanisms. It acts as a potent scavenger of ROS, directly neutralizing free radicals and attenuating oxidative stress [66]. In our study, crocin significantly elevated SOD activity, consistent with previous findings showing its ability to upregulate antioxidant enzymes including catalase and glutathione peroxidase [61,67–69]. This antioxidative action is crucial in preventing β-cell dysfunction and insulin resistance—two major pathological features of diabetes [70].

Furthermore, crocin activates insulin signaling pathways by enhancing AKT phosphorylation, thereby promoting glucose uptake into muscle and adipose tissue via GLUT4 translocation. This effect has been demonstrated in various diabetic models and was reaffirmed in our study through the observed upregulation of AKT gene expression in the liver [71]. Crocin may also improve β-cell mass and stimulate insulin secretion, contributing further to its hypoglycemic effects [72,73].

In addition to its antioxidant and insulin-sensitizing effects, crocin exhibits strong anti-inflammatory properties, which contribute to its protective role against diabetes-induced organ damage. Crocin’s ability to reduce doxorubicin-induced hepatotoxicity through attenuation of lipid peroxidation and inflammation, findings consistent with our histopathological observations in diabetic liver tissue [74].

Interestingly, both *C. lancifolius* extract and crocin exerted convergent effects in enhancing SOD activity and AKT gene expression, suggesting overlapping but potentially complementary mechanisms. While crocin’s molecular pathways are well-characterized in the literature, our study adds novel mechanistic insights into the bioactivity of *C. lancifolius*, which remains relatively underexplored. The induction of AKT and SOD gene expression by *C. lancifolius* provides compelling evidence for its potential use as an adjunct therapy in diabetes management.

Overall, this study demonstrates that *C. lancifolius* extract and crocin mitigate hyperglycemia, oxidative stress, and tissue injury in diabetic rats via activation of the PI3K/AKT signaling axis and enhancement of endogenous antioxidant defenses. These findings align with previous mechanistic insights [63,66,71,75] and provide new evidence supporting *C. lancifolius* as a promising natural candidate for antidiabetic therapy. The histopathological protection observed in key metabolic organs strengthens the translational relevance of both compounds.

In our study, both treatments restored normal tissue histoarchitecture, upregulated AKT protein expression, and enhanced SOD enzyme activity, confirming their antioxidant and insulin-sensitizing effects. These findings are in alignment with improved glucose handling and organ protection, which are consistent with earlier reports for crocin [76,77] but now demonstrated here for *C. lancifolius* for the first time.

The observed activation of the AKT pathway suggests that both *C. lancifolius* extract and crocin modulate insulin signaling, possibly via interaction with the insulin receptor or its downstream effectors. While direct interaction with insulin receptors was not evaluated in this study, the upregulation of AKT indicates activation of the PI3K/AKT pathway, a key downstream event in insulin receptor signaling. Future studies are warranted to investigate the precise mechanism of interaction, including whether these agents enhance insulin receptor sensitivity or mimic insulin action via alternative upstream signals, such as AMPK or IGF-1 receptor pathways. Similar insulin-independent pathways have been proposed for crocin in prior research [78], and our data suggest *C. lancifolius* may function through a comparable mechanism. In addition, future research should focus on isolating specific bioactive components within *C. lancifolius*, exploring dose-response relationships, and conducting clinical investigations to validate efficacy in human subjects. Integrating such plant-derived therapies into diabetes management could offer safer, multi-targeted alternatives to synthetic drugs.

## 5 Conclusion

Crocin and *Conocarpus lancifolius* extract exhibit significant therapeutic potential in the management of diabetes, offering multiple benefits including improved glycemic control, protection of vital organs, and modulation of insulin signaling pathways. This study highlights their ability to enhance antioxidant defenses, preserve pancreatic β-cell function, and increase insulin sensitivity, as evidenced by reductions in fasting glucose levels and improvements in AKT gene expression.

Histopathological analysis further confirmed the protective effects of both treatments on the pancreas, liver, kidneys, and heart, demonstrating their capacity to restore normal tissue architecture and function. These protective effects are largely attributed to the antioxidant properties of crocin and *C. lancifolius*, which counteract oxidative stress—a major contributor to diabetic complications. Notably, crocin normalized the activity of key antioxidant enzymes such as SOD, reinforcing its role in oxidative stress mitigation.

While the current findings are encouraging, further research is essential, particularly studies focusing on the molecular mechanisms of action, dose-response relationships, and clinical trials in human subjects. Crocin, with its natural origin and favorable safety profile, presents an attractive adjunct to existing antidiabetic therapies and may improve patient adherence in the long term. Likewise, *C. lancifolius* shows promise as a valuable source of bioactive phytochemicals, warranting continued investigation for the development of novel plant-based therapeutic agents.

In summary, both crocin and *C. lancifolius* extract hold substantial promise as complementary approaches to diabetes management, with the potential to enhance treatment outcomes and patient quality of life.

## Supporting information

S1 Fig Western blot results – first three repeats. (TIF)

S2 Fig Western blot results – second three repeats. (TIF)

S3 Fig Western blot results – third three repeats. (TIF)

S4 Fig Western blot results – all three repeats combined. (TIF)
