# Supplementary figures and images for: Effects of Conocarpus lancifolius and crocin on superoxide dismutase and protein kinase B genes and protein expressions in diabetic rats
Source: PLoS One. 2025 Jun 26;20(6):e0326676. doi: 10.1371/journal.pone.0326676 (PMC12200675; doi:10.1371/journal.pone.0326676)

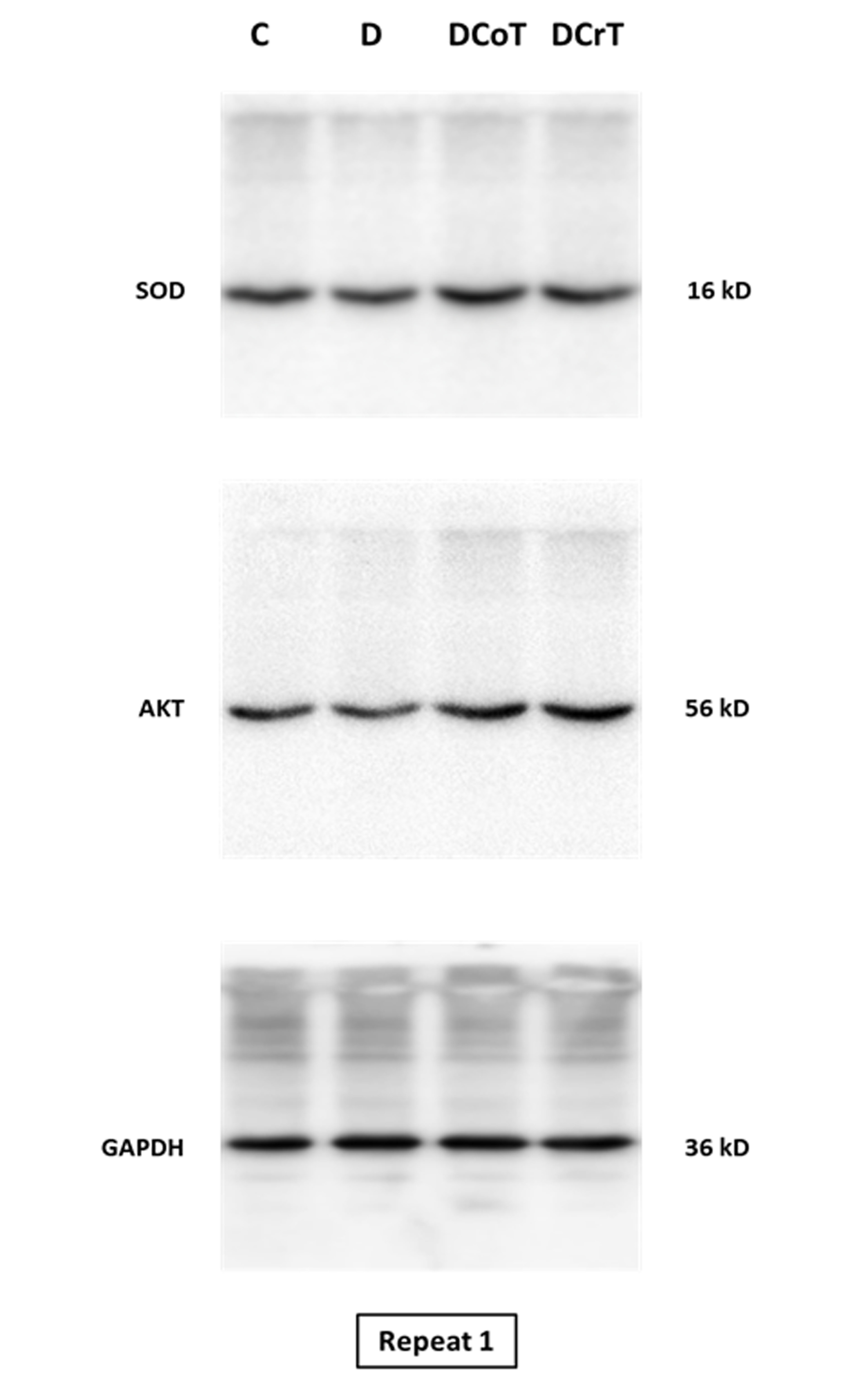

Supplement: S1 Fig — (TIF) [file pone.0326676.s001.tif]

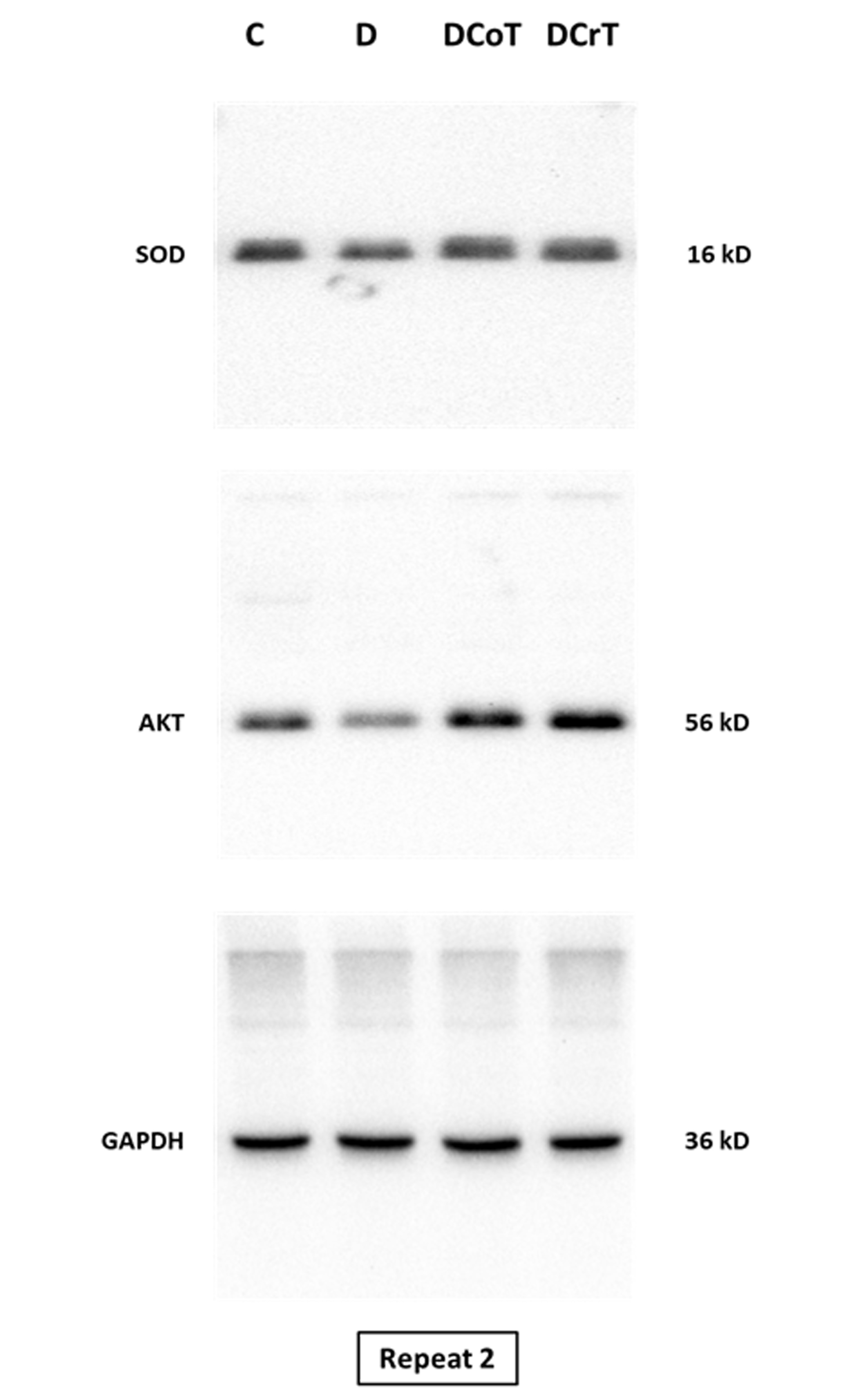

Supplement: S2 Fig — (TIF) [file pone.0326676.s002.tif]

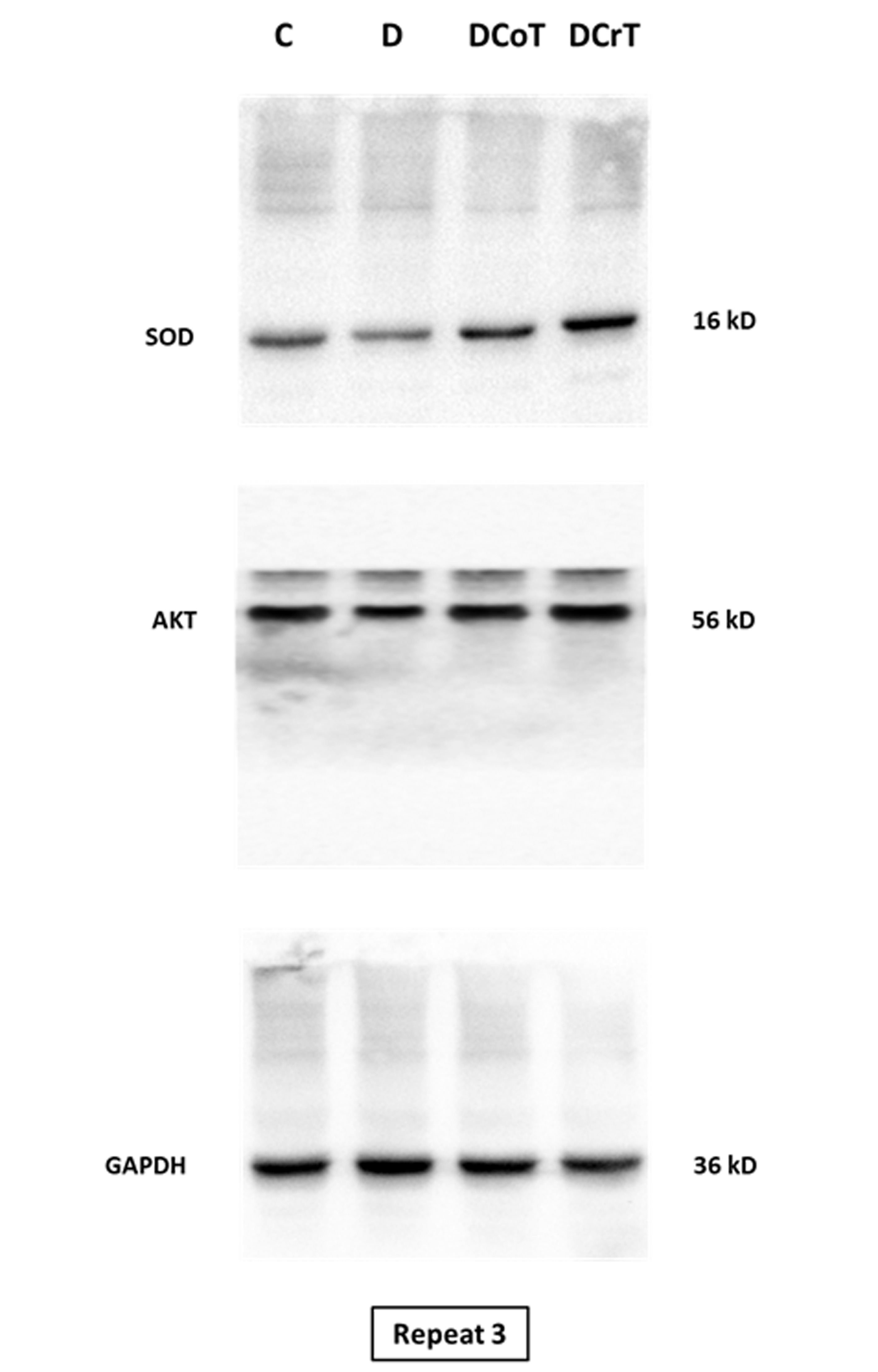

Supplement: S3 Fig — (TIF) [file pone.0326676.s003.tif]

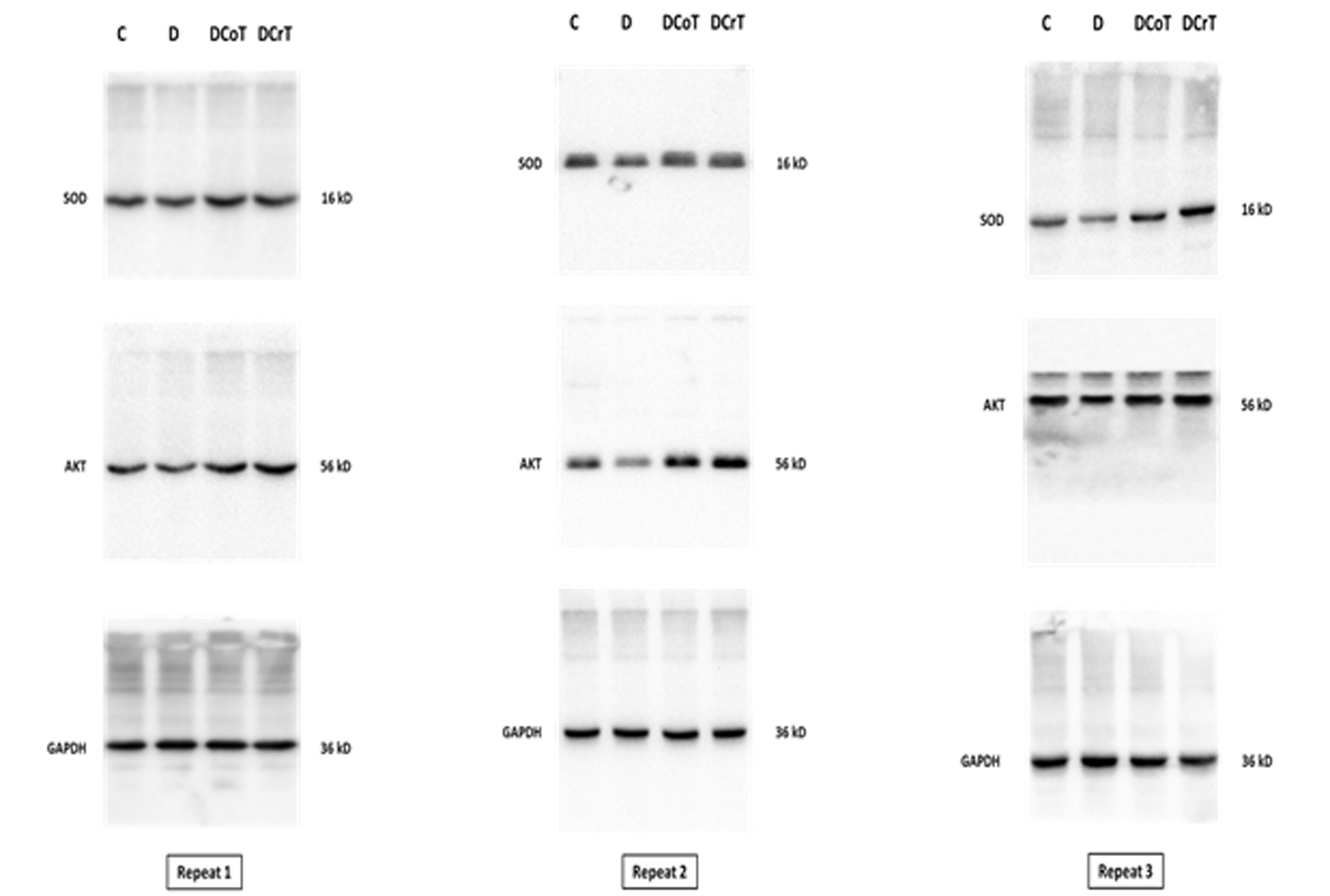

Supplement: S4 Fig — (TIF) [file pone.0326676.s004.tif]
